# Prominent efficacy and good safety of sequential CD19 and CD22 CAR-T therapy in relapsed/refractory adult B-cell acute lymphoblastic leukemia

**DOI:** 10.1186/s40164-024-00593-5

**Published:** 2025-01-03

**Authors:** Tingting Yang, Yetian Dong, Mingming Zhang, Jingjing Feng, Shan Fu, Pingnan Xiao, Ruimin Hong, Huijun Xu, Jiazhen Cui, Simao Huang, Guoqing Wei, Delin Kong, Jia Geng, Alex H. Chang, He Huang, Yongxian Hu

**Affiliations:** 1 Bone Marrow Transplantation Center of The First Affiliated Hospital Liangzhu Laboratory, Zhejiang University School of Medicine, No. 79 Qingchun Road, Hangzhou, Zhejiang China; 2 Institute of Hematology, Zhejiang University, Hangzhou, Zhejiang China; 3 Zhejiang Province Engineering Laboratory for Stem Cell and Immunity Therapy, Hangzhou, Zhejiang China; 4 Department of Radiology of The First Affiliated Hospital Liangzhu Laboratory, Zhejiang University School of Medicine, Hangzhou, Zhejiang China; 5 Engineering Research Center of Gene Technology, Ministry of Education, Institute of Genetics, School of Life Sciences, Fudan University, Shanghai, China; 6 Shanghai YaKe Biotechnology Ltd., Shanghai, China

**Keywords:** Relapsed/refractory B-cell acute lymphoblastic leukemia, Chimeric antigen receptor, Sequential therapy

## Abstract

**Background:**

Sequential CD19 and CD22 chimeric antigen receptor (CAR)-T cell therapy offers a promising approach to antigen-loss relapse in relapsed/refractory (R/R) B-cell acute lymphoblastic leukemia (B-ALL); however, research in adults remains limited.

**Methods:**

This study aimed to evaluate the efficacy and safety of sequential CD19 and CD22 CAR-T cell therapy in adult patients with R/R B-ALL between November 2020 and November 2023 (ChiCTR2100053871). Key endpoints included the adverse event incidence, overall survival (OS), and leukemia-free survival (LFS).

**Results:**

Twenty-three patients with a median age of 58.1 years (range 25.9–75.0) were enrolled. High-risk cytogenetic and genomic aberrations were identified in 43.5% of patients, and five patients had baseline extramedullary disease (EMD). The median interval between the two infusions was 3.8 months. Grade ≥ 3 hematological adverse events occurred at comparable rates after both infusions. Cytokine release syndrome was observed in 78.3% and 39.1% of patients after CD19 and CD22 CAR-T therapy, respectively. Two patients experienced grade 2 immune effector cell-associated neurotoxicity syndrome (ICANS) during CD19 CAR-T, and no ICANS was reported during CD22 CAR-T. The median OS was not reached with a median follow-up of 19.4 months (range 8.7–45.6), while the median LFS was 20.8 months. OS and LFS rates were 91.3% and 67.1% at 1 year and 58.6% and 47.0% at 2 years, respectively. Eight patients experienced relapse, with the cumulative incidence of relapse being 28.6% at 1 year and 42.5% at 2 years. Higher baseline leukemia burden (≥ 64% bone marrow blasts) and the presence of EMD were significant risk factors for inferior OS and LFS, respectively.

**Conclusions:**

Sequential CAR-T cell therapy demonstrated durable efficacy and a manageable safety profile in R/R B-ALL, providing a viable option to address antigen-loss relapse and improve long-term outcomes in high-risk adult patients.

**Supplementary Information:**

The online version contains supplementary material available at 10.1186/s40164-024-00593-5.

## Background

Chimeric antigen receptor (CAR)-T cell therapy has revolutionized the treatment landscape for relapsed/refractory (R/R) B-cell acute lymphoblastic leukemia (B-ALL), achieving remarkable clinical responses of 70% to 90% [1, 2]. Despite these impressive initial response rates, 20–50% of responders experience relapse within 6 months [3–6]. Antigen downregulation or loss, occurring through various mechanisms, is the primary obstacle to durable responses and accounts for up to 60% of all relapse cases [7–10]. Allogeneic hematopoietic stem cell transplantation (allo-HSCT) as a consolidative therapy may not be feasible for patients with poor physical condition, severe infections, or organ dysfunction. Additionally, severe complications and the potential elimination of residual CAR-T cells limit its applicability.

To overcome these challenges, researchers are increasingly focusing on dual- or multi-antigen-targeting CAR-T cell strategies. CD22 has emerged as an outstanding target for CAR-T therapy, demonstrating therapeutic effects comparable to those of CD19 CAR-T therapy in preclinical and clinical studies. CD22 CAR-T cell therapy can also eliminate leukemic cells that have lost CD19 expression, thereby addressing a key relapse mechanism [11]. A recent study by Pan et al*.* confirmed that sequential CD19 and CD22 CAR-T cell therapy could deepen responses and improve leukemia-free survival (LFS) in pediatric B-ALL [12]. However, clinical trials evaluating the benefits of this sequential therapy in adults and older patients are limited and require further investigation. Thus, in this study, we aimed to investigate the efficacy and safety of sequential CD19 and CD22 CAR-T cell therapy in 23 adult patients with R/R B-ALL who were ineligible for or unwilling to undergo allo-HSCT.

## Methods

### Patients

This study was conducted at the First Affiliated Hospital of Zhejiang University from November 2020 to November 2023 and registered at www.chictr.org.cn (ChiCTR2100053871). It was approved by the Ethics Review Committee of the First Affiliated Hospital of Zhejiang University and conducted in accordance with the Declaration of Helsinki. Informed consent was obtained from all patients or their legal guardians before enrollment. Details of the inclusion and exclusion criteria for patients are provided in the supplementary methods.

### CAR-T cell infusions

CD19 and CD22 CAR-T cells were manufactured according to the manufacturer’s standard, as previously reported (supplementary methods) [13, 14]. Prior to each CAR-T cell infusion, a lymphodepletion regimen consisting of fludarabine (30 mg/m^2^/day) and cyclophosphamide (500 mg/m^2^/day) was administered on days -5, -4, and -3. The second round, involving CD22 CAR-T cell infusion, was administered at least 1 month after the initial CD19 CAR-T cell infusion. The exact interval between the two infusions was based on the patient’s disease status, resolution of adverse events (AEs), and absence of infections. Patients were closely monitored to ensure that all side effects from the first infusion subsided and that they were in stable condition before proceeding with the second infusion. Patients who did not achieve complete remission (CR) after the first infusion or those with unresolved grade ≥ 3 AEs, were ineligible for the second infusion.

### Assessment and management of AEs

AEs following CAR-T cell infusion were continuously monitored. Cytokine release syndrome (CRS) and neurotoxicity were graded based on the American Society for Transplantation and Cellular Therapy criteria. Other AEs were evaluated according to the National Cancer Institute Common Terminology Criteria for Adverse Events, version 5.0. B-cell aplasia (BCA) was defined as less than 3% CD19/CD22-positive lymphocytes in peripheral blood (PB) or bone marrow (BM). Regular follow-up and laboratory tests were conducted to assess efficacy and detect late-onset AEs.

### Kinetics of CAR-T cells

Pharmacokinetic evaluation included the expansion and persistence of CD19 and CD22 CAR-T cells in the PB, and if needed, in the cerebrospinal fluid and BM. In vivo expansion of CD19 and CD22 CAR-T cells was measured using multiparameter flow cytometry. The flow cytometry protocol for detecting CAR-T cells included the following markers and antibodies: APC-labeled anti-human CD45 detected with the HI30 clone (Cat# 304037, BioLegend); PE-Cy7-labeled anti-human CD3 detected with the UCHT1 clone (Cat# 300420, BioLegend); FITC-labeled CD19 (Yake company); FITC-labeled CD22 (Yake company); PE-labeled anti-human CD8 (Cat# 301051, BioLegend); and PerCP-Cy5.5-labeled anti-human CD4 (Cat# 317418, BioLegend). T-cell and B-cell monitoring included BV510-labeled anti-human CD45 (Cat# 583204, BD Biosciences), APC-labeled anti-human CD3 (Cat# 300439, BioLegend), FITC-labeled CD19 (Cat# 302206, BioLegend), and PE-labeled CD22 (Cat# 302506, BioLegend).

### Outcome assessments

Disease status and treatment responses were assessed after each CAR-T cell infusion according to the National Comprehensive Cancer Network (NCCN) guidelines, version 3, 2020. High-risk features included complex karyotypes, hypodiploidy, specific rearrangements like t(v;11q23), t(4;11) and KMT2A rearrangements, BCR-ABL1 fusion gene, IKZF1 alteration, TP53 mutation, intrachromosomal amplification of chromosome 21 (iAMP21). CR was defined as having less than 5% blasts in the BM, the absence of circulating blasts, and no evidence of extramedullary disease (EMD) involvement. Minimal residual disease (MRD) negativity was established as less than 0.01% BM blasts detected by flow cytometry. Leukemia relapse referred to the recurrence of ≥ 5% leukemic blasts, whether in PB, BM, or the development of EMD infiltration after CR.

Overall survival (OS) was defined as the time from the first CD19 CAR-T cell infusion to death from any cause or last follow-up. LFS was defined as the time from the first CD19 CAR-T cell infusion to the earliest leukemia relapse or death. The cumulative incidence of relapse (CIR) was calculated from the date of the first CD19 CAR-T cell infusion until leukemia relapse occurred.

### Statistical analysis

Data were analyzed using descriptive statistics for demographic and baseline characteristics. Cytokine and CAR-T cell levels were compared using the t-test or Mann–Whitney U test. Categorical variables were compared using the chi-squared or Fisher’s exact test. Time-to-event analyses, including OS and LFS, were performed using the Kaplan–Meier methods. CIR was evaluated using a competing risk model and compared by the Fine-Gray test, considering non-relapse mortality as a competing risk. Cox proportional hazards regression models for univariate and multivariate analyses were performed to identify independent prognostic factors for OS and LFS. Significant variables (*P* < 0.2) in the univariate analyses were included into the multivariate analyses with a backward stepwise selection model. Statistical significance was determined using a two-sided *P*-value of < 0.05. All statistical analyses were performed using R software (version 4.2.2) and IBM SPSS Statistics (version 26). The cut-off date for follow-up was August 18, 2024.

## Results

### Patient characteristics

**Fig. 1:**
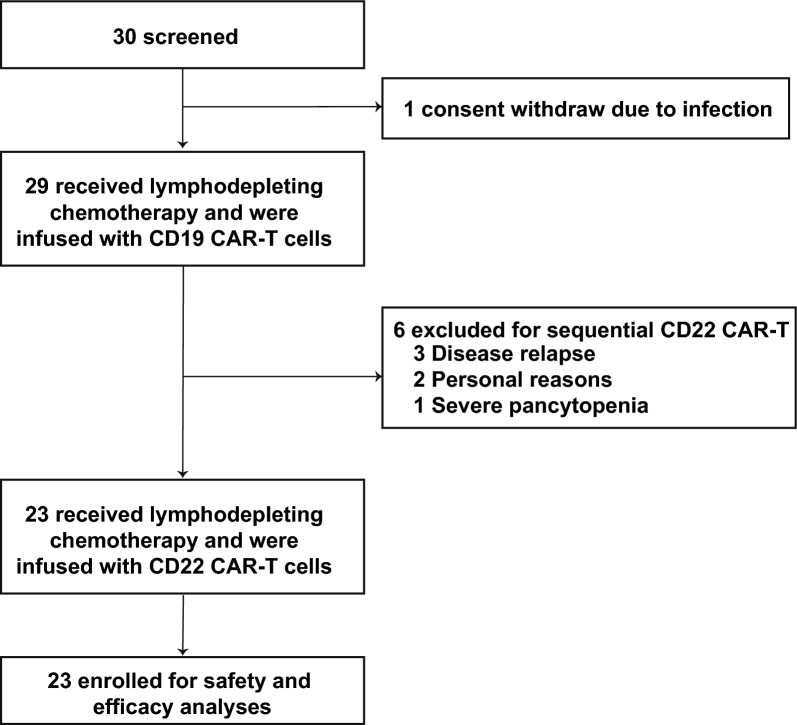
Diagram of the sequential treatment procedure

Between November 2020 and November 2023, 30 patients were screened, and 23 were enrolled to receive sequential CD19 and CD22 CAR-T cell therapy (Fig. 1). One patient withdrew due to a severe infection. Six patients discontinued after the CD19 CAR-T cell infusion and did not receive CD22 CAR-T cells due to disease relapse (n = 3), personal reasons (n = 2), or severe AE (n = 1). The baseline characteristics of patients are summarized in Table 1. Of the enrolled patients, 12 (52.2%) were male, and 11 (47.8%) were female, with a median age of 58.1 years (range, 25.9–75.0). High-risk cytogenetic and genomic aberrations were detected in 10 patients (43.5%) (Table S1). BCR-ABL fusion genes were detected in four patients, complex chromosome karyotypes in three patients, IKZF1 alterations in two patients, and TP53 mutation in one patient. Nineteen patients (82.6%) had undergone more than three prior lines of therapy, seven had previously undergone allo-HSCT, and one had previously received dual CD19 and CD22 CAR-T therapy. At enrollment, five patients had EMD: three with isolated diffuse EMD, one with concurrent BM and diffuse EMD involvement, and one with BM and CNS involvement (Table S2). Eight patients received bridging therapy, including bispecific T-cell engager (BITE, n = 6) and chemotherapy (n = 2), to reduce circulating blasts before lymphodepletion (Table S3).

**Table 1:** Baseline characteristics of all enrolled patients

Characteristic	All treated patients (N = 23)
Age (years), median (range)	58.1 (25.9–75.0)
Sex, n (%)	
Male	12 (52.2)
Female	11 (47.8)
Poor-risk cytogenetics, n (%)	10 (43.5)
BCR/ABL positive, n (%)	4 (17.4)
Complex chromosome karyotype, n (%)	3 (13.0)
Prior lines of therapy, median (range)	6.5 (2–12)
Previous allogeneic HSCT, n (%)	7 (30.4)
Extramedullary disease, n (%)	5 (21.7)
Bridging therapy at baseline, n (%)	
Chemotherapy	2 (8.7)
BITE	6 (26.1)
BM MRD before CAR T-cell therapy	
< 1%	10 (43.5%)
1% to < 15%	4 (17.4%)
≥ 15%	9 (39.1%)

BITE: bispecific T-cell engager; BM: bone marrow; CAR-T: chimeric antigen receptor-T; HSCT: hematopoietic stem cell transplantation; MRD: minimal residual disease

### Characteristics of the infused CAR-T cell product

The median transduction efficiency was 59.7% (range, 10.6–77.9%) for CD19 CAR-T cells and 48.1% (range, 20.7–70.0%) for CD22 CAR-T cells. The median infusion doses were 2.1 × 10^6^ cells/kg (range 0.8–3.6 × 10^6^) for CD19 CAR-T cells and 2.1 × 10^6^ cells/kg (range 1.1–3.0 × 10^6^) for CD22 CAR-T cells. Among the seven patients with a history of allo-HSCT, five received donor-derived CD19 and CD22 CAR-T products (four from haploidentical donors and one from a matched sibling donor), while the remaining two received autologous CAR-T products due to the unavailability of donor T cells (Table S4). A comparison of the two CAR-T cell types revealed that CD19 CAR-T cells had a significantly higher transduction efficiency (*P* = 0.01), CD4 CAR-T/CAR-T ratio (*P* < 0.001), and CD4/CD8 CAR-T ratio (*P* = 0.02), whereas CD22 CAR-T cells showed higher CD8 CAR-T/CAR-T ratios (*P* < 0.001) than did CD19 CAR-T cells (Table S5). No significant differences in CAR-T product characteristics were observed between patients who achieved ongoing remission and those who experienced relapse (Table S6).

### Safety

All AEs associated with CD19 and CD22 CAR-T cells are presented in Table S7. No treatment-related deaths occurred. Following CD19 CAR-T therapy, hematologic toxicities were the most common grade ≥ 3 AEs, including lymphopenia (100%), neutropenia (91.3%), anemia (52.2%), and thrombocytopenia (52.2%) (Fig. S1A). Among patients with grade 3 or higher cytopenia, 69.6% recovered to grade ≤ 2 lymphopenia, 81.0% to grade ≤ 2 neutropenia, 41.7% to grade 2 anemia, and 50% to grade 2 thrombocytopenia within 1 month after infusion. The median lymphocyte count before the second lymphodepletion was 1.1 × 10^9^/L (range 0.5–5.7 × 10^9^/L), similar to the level before the first lymphodepletion (1.1 × 10^9^/L; range 0.5–4.2 × 10^9^/L) (Fig. S1B). During CD22 CAR-T therapy, grade 3 or 4 lymphocytopenia occurred in all patients, neutropenia in 87.0%, anemia in 21.7%, and thrombocytopenia in 52.2%. One month after CD22 CAR-T infusion, among patients with grade 3 or higher cytopenia, recovery to grade ≤ 2 lymphopenia was observed in 82.6%, neutropenia in 80%, anemia in 40%, and thrombocytopenia in 50%. Red blood cell transfusions were administered to six patients (26.1%) during CD19 CAR-T and three (13.0%) during CD22 CAR-T. Platelet transfusions were administered to five patients (21.7%) following CD19 CAR-T and two (8.7%) following CD22 CAR-T. Infections occurred in seven patients (30.4%) during the first infusion, including pneumonia (n = 4), intestinal infection (n = 1), urinary tract infection (n = 1), and bloodstream infection (n = 1). During the second infusion, six patients (26.1%) developed infections, including pneumonia (n = 3), intestinal infection (n = 2), and bloodstream infection (n = 1). All infections were successfully resolved. After CD19 CAR-T cell infusion, no patient experienced Epstein-Barr virus or cytomegalovirus (CMV) reactivation. After the CD22 CAR-T infusion, only one patient developed CMV reactivation.

CRS occurred in 18 patients (78.3%) during CD19 CAR-T cell therapy, including seven (30.4%) with grade 1, nine (39.1%) with grade 2, and two (8.7%) with grade 3 CRS. The primary manifestation of CRS was fever, with only one patient exhibiting localized facial swelling, which was considered focal CRS. The median onset and duration of CRS were 4 days (range 1–10) and 6 days (range 2–13), respectively. All grade 1 CRS cases and two grade 2 CRS cases were managed with symptomatic treatment; two grade 2 CRS cases received only steroids, while five grade 2 CRS cases and two grade 3 CRS cases were treated with steroids and tocilizumab. After CD22 CAR-T, nine patients (39.1%) experienced CRS (grade 1, n = 3; grade 2, n = 6), with a median onset and duration of 1 day (range 1–5) and 5 days (range 3–14), respectively. No CRS of grade 3 or worse was documented. One patient received steroids and tocilizumab, two received steroids alone, and six received symptomatic treatment. In both infusions, patients with higher-grade CRS (≥ grade 2) had significantly higher peak concentrations of serum interleukin-6 (IL-6), IL-10, interferon-γ, and C-reactive protein than did those with low-grade CRS or without CRS (grade 0–1 CRS) (Fig. S2). The IL-6 levels in patients with CRS ≥ 2 during CD19 CAR-T therapy were significantly higher than those in patients with CRS ≥ 2 during CD22 CAR-T therapy (*P* = 0.03), while no significant differences were observed for other cytokines (Fig. S3). Baseline BM disease burden was comparable between patients with grade ≥ 2 CRS and those with grade 0–1 CRS during CD19 CAR-T infusion (*P* = 0.12) (Fig. S4). Two patients developed grade 2 immune effector cell-associated neurotoxicity syndrome (ICANS), mainly manifesting with drowsiness, confusion, sluggish responses, and incoherent answers. One of whom had CNS leukemia (CNSL) and developed ICANS with concomitant grade 2 CRS. The onset of ICANS was observed at 6 days and 16 days post-infusion, with both cases lasting for 2 days each. Symptoms resolved without clinical sequelae. No ICANS was reported during CD22 CAR-T cell therapy.

### Efficacy

**Fig. 2:**
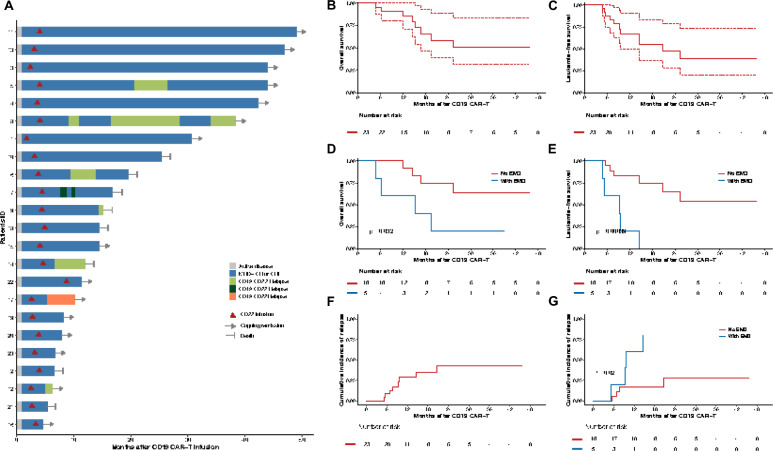
Clinical outcomes. **A** Swimmer plot showing the duration of response and survival outcomes post-infusion for all treated patients (n = 23). CR/CRi, complete remission/complete remission with incomplete hematologic recovery; MRD^−^, minimal residual disease-negative. **B**, **C** Kaplan–Meier estimates of overall survival (OS, **B**) and leukemia-free survival (LFS, **C**) in all patients. **D**, **E** Kaplan–Meier estimates of OS (**D**) and LFS (**E**) in patients without extramedullary disease (EMD) (n = 18) versus those with EMD (n = 5). **F** Cumulative incidence of relapse in all patients. **G** Cumulative incidence of relapse in patients without EMD versus those with EMD

Following CD19 CAR-T cell therapy, the overall response rate was 100%, with all patients achieving MRD-negative (MRD^−^) CR. After sequential infusions, two patients received one course of sintilimab as consolidation therapy, and three BCR-ABL-positive patients continued taking tyrosine kinase inhibitors. With a median follow-up of 19.4 months (range 8.7–45.6), 15 patients (65.2%) were alive, and 14 (60.9%) remained alive in molecular remission without further treatment (Fig. 2A). Eight patients died during follow-up owing to infectious shock (n = 4), disease progression (n = 3), and HSCT-associated complications (n = 1). The median OS was not reached, and the median LFS was 20.8 months. The OS rates were 91.3% (95% confidence interval [CI], 80.5–100%) at 1 year and 58.6% (95% CI 38.7–88.8%) at 2 years (Fig. 2B). The 1-year and 2-year LFS rates were 67.1% (95% CI 49.5–90.9%) and 47.0% (95% CI 28.2–78.4%), respectively (Fig. 2C).

Eight patients experienced leukemia relapse: six (75.0%) with CD19^+^CD22^+^ relapse, one (12.5%) with CD19^−^CD22^+^ relapse, and one (12.5%) with CD19^−^CD22^−^ relapse (Fig. S5A and Table S8). There were six cases of BM relapse, one of isolated extramedullary relapse, and one of coexisting BM and CNSL relapse (Fig. S5B). The median time to relapse following CD19 and CD22 CAR-T cell infusion was 8.5 months (range 5.1–20.8) and 4.2 months (range 2.1–16.7), respectively. CIR at 1 year and 2 years were 28.6% and 42.5%, respectively (Fig. 2F).

Among the five patients with EMD involvement, the extramedullary lesions achieved CR following the initial CD19 CAR-T infusion. After sequential infusions, of the three patients with isolated EMD, one patient succumbed to septic shock 1.3 months after CD22 CAR-T cell infusion. Another patient experienced isolated EMD relapse, with new lesions identified in the kidneys, heart, and tonsils. One patient experienced only BM relapse. In patients with BM and extramedullary involvement, one had BM relapse, while another had both BM and CNS relapse. The median time to relapse was 9.4 months (range 5.1–14.4) following CD19 CAR-T cell infusion and 5.4 months (range 2.6–10) following CD22 CAR-T cell infusion. Patients with EMD had inferior OS (*P* = 0.032) and LFS (*P* < 0.001) (Fig. 2D–E). A significantly higher CIR was observed in patients with EMD compared to those without EMD (*P* = 0.02) (Fig. 2G).

### Kinetics of CAR-T cells

**Fig. 3:**
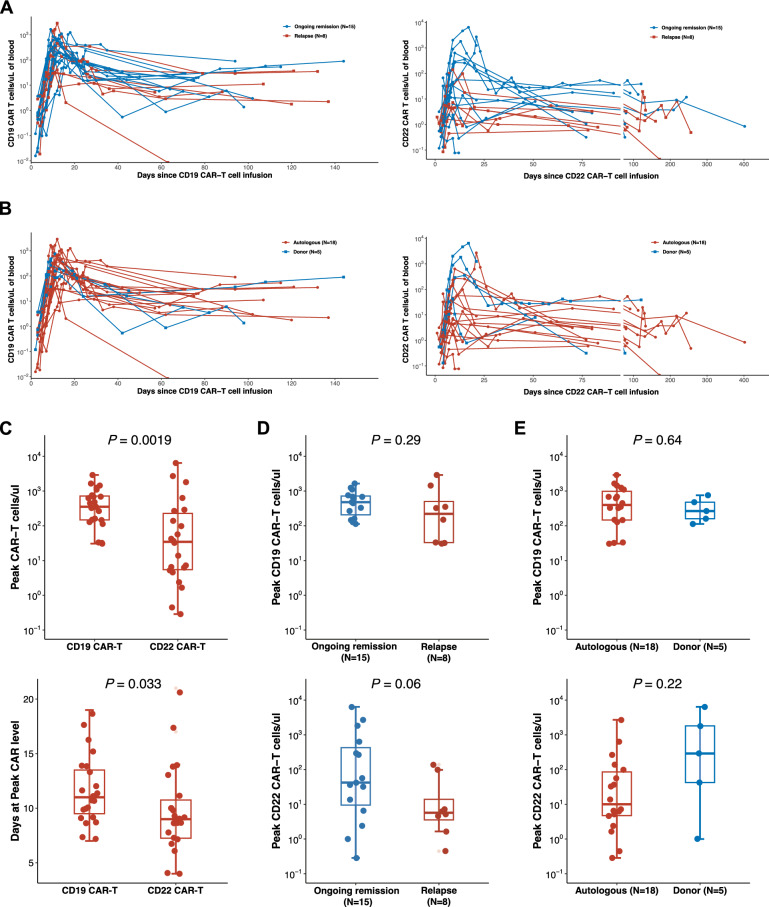
Dynamics of CAR-T cell expansion and comparison of peak CAR-T levels. **A** Expansion of CAR-T cells over time post-infusion in all patients. **B** CAR-T cell expansion between autologous and donor-derived CAR-T cells. **C** Peak CAR-T cell levels and the number of days to reach peak expansion. **D** Peak CD19 CAR-T cell levels (top) and peak CD22 CAR-T cell levels (bottom) between patients with ongoing remission and those who relapsed. **E** Peak CD19 CAR-T cell levels (top) and peak CD22 CAR-T cell levels (bottom) between autologous and donor-derived CAR-T cells

The full kinetic profiles of circulating CAR-T cell levels, measured by flow cytometry in patients over time, are shown in Fig. 3A and B. CAR-T cell expansion was observed in all patients throughout the treatment cycles. During the first infusion, rapid CAR T-cell expansion was observed with a median time to peak of 11 days (range, 7–19) and a median peak level of 353.2 cells/μL (range, 30.6–2902.1) (Fig. 3C). Prior to CD22 CAR-T therapy, the median level of CD19 CAR-T cells was 1.8 cells/μL (range, 0–89.8), and the median percentage of CD19 CAR-T cells in PB lymphocytes was 0.9% (range, 0–17.5%) (Fig. S6A and B). During the second infusion, the median peak CAR T-cell expansion occurred on day 9 (range, 4–21), with a median peak level of 34.5 cells/μL (range, 0.8–6415.4) (Fig. 3C). Despite the comparable infusion doses of CD19 and CD22 CAR-T cells, the peak CAR-T cell levels were significantly higher in the first cycle than in the second cycle, indicating a more robust initial expansion. Peak CD22 CAR-T cell levels were lower in patients who experienced relapse than in those in remission (*P* = 0.06), and peak CD19 CAR-T cell levels showed no significant difference between the two groups (*P* = 0.29) (Fig. 3D). No significant differences in both CAR-T cell expansion were observed among the different CAR-T product sources (Fig. 3E). Further investigation of the correlation between EMD and peak CAR T-cell expansion revealed no significant correlation with either CAR-T infusion (Fig. S7). Among the 8 relapsed patients, CAR-T cells were undetectable by flow cytometry.

### BCA

BCA in the PB or BM was observed in all patients 1 month after CD19 CAR-T therapy. Before the second CAR-T cell infusion, the median B-cell percentage in peripheral lymphocytes was 2.1% (range, 0.15–26.1%) (Fig. S6C). Twelve patients had sustained BCA, while eleven had B-cell recovery with a median percentage of 6.5% (range, 3.3–26.1%) of peripheral lymphocytes. Of the 14 patients with durable responses, three (21.4%) maintained BCA for longer than 6 months following CD22 CAR-T cell infusion. The remaining 11 patients experienced B-cell recovery with a median time of 2.8 months (range, 1.2–5.8) after CD22 CAR-T infusion. Among the eight patients with relapse, loss of BCA preceding relapse was observed in three cases (37.5%), concurrent loss of BCA and relapse in four cases (50%), and ongoing BCA in one case (12.5%). For MRD^−^CR patients with follow-up exceeding 6 months after CD22 infusion, immunoglobulin recovery at the cut-off date was observed in six cases (75.0%) for IgM, five (62.5%) for IgA, and five (62.5%) for IgG.

### Multivariate analyses

Multivariate analyses for clinical outcomes were conducted (Table 2). A higher leukemia burden at baseline (≥ 64% blast cells in BM) was an independent prognostic factor correlated with shorter OS (hazard ratio [HR], 5.9; 95% CI 1.3–26.7, *P* = 0.021). Multivariable analysis for LFS revealed that the presence of EMD at baseline contributed significantly to inferior LFS (HR, 8.9; 95% CI 2.1–37.7; *P* = 0.003). Conversely, patient age, sex, risk stratification, prior lines of therapy, interval between the two CAR-T infusions, and loss of BCA before CD22 CAR-T cell infusion were not predictive of OS or LFS.

**Table 2:** Univariate and multivariate analysis of factors influencing OS and LFS

	OS				LFS			
	Univariate analysis		Multivariate analysis		Univariate analysis		Multivariate analysis	
	HR (95% CI)	*P*	HR (95% CI)	*P*	HR (95% CI)	*P*	HR (95% CI)	*P*
Age (years)								
< 58 vs ≥ 58	0.7 (0.17–2.9)	0.62			1.6 (0.46–5.5)	0.55		
Sex								
Female vs Male	1.2 (0.31–5)	0.76			0.5 (0.15–1.7)	0.27		
BCR/ABL-positive								
No vs Yes	0.6 (0.07–4.9)	0.63			0.36 (0.05–2.9)	0.34		
NCCN risk stratification								
Good vs Poor	0.58 (0.14–2.4)	0.45			0.64(0.19–2.2)	0.48		
Lines of prior therapy								
< 8 vs ≥ 8	0.87 (0.2–3.7)	0.85			0.53 (0.14–2.1)	0.36		
Prior HSCT								
No vs Yes	1.2 (0.23–5.9)	0.86			0.58 (0.12–2.7)	0.48		
BM blasts at baseline (%)								
< 64 vs ≥ 64	5.9 (1.3–27)	0.021	5.9 (1.3–26.7)	0.021	4 (1.1–15)	0.04	2.9 (0.64–12.6)	0.17
EMD at baseline								
No vs Yes	4.1 (1–16)	0.048	1.59 (0.29–8.8)	0.59	8.9 (2.1–38)	0.0031	8.9 (2.1–37.7)	0.003
Interval between two CAR-T infusions (months)								
< 3.6 vs ≥ 3.6	1.2 (0.3–5.1)	0.8			1.3 (0.4–4.9)	0.59		
Loss of BCA before CD22 CAR-T infusion								
No vs Yes	0.93 (0.23–3.7)	0.91			2.2 (0.65–7.6)	0.2	1.3 (0.29–6.4)	0.71

BM: bone Marrow; BCA: B-cell aplasia; EMD: extramedullary disease; HSCT: hematopoietic stem cell transplantation; LFS: leukemia-free survival; NCCN: National Comprehensive Cancer Network; OS: overall survival

## Discussion

Remission durability after single-antigen targeted CAR-T cell therapy is often compromised by antigen modulation, which can be addressed with combinatorial targeting strategies. Clinical evidence has demonstrated that dual-antigen CAR-T cell therapy, including simultaneous and sequential infusions, can exhibit synergistic effects, optimizing response rates and thus enhancing therapeutic efficacy over single-antigen targeting [8, 15–23]. A comparative study of single-target (CD19) versus dual-target (tandem or sequential CD19/CD22) CAR-T therapy in R/R B-ALL found no difference in efficacy between the tandem and sequential CD19/CD22 therapies; however, both were superior to the single-target CAR-T therapy [24]. Pan et al*.* in a phase 2 study also demonstrated superior OS and LFS with sequential CD19/CD22 CAR-T therapy in pediatric R/R B-ALL [12]. Our study extended these findings to a predominantly elderly patient population, confirming the efficacy and safety of sequential CD19/CD22 CAR-T cell therapy. OS and LFS rates at 1 year exceeded 50–74% and 10–58%, respectively, observed in previous single-target (CD19 or CD22) CAR-T cell studies, reported by our center and other institutions [6, 25–27]. Notably, our previous data revealed dismal outcomes and high relapse rates for patients without concurrent consolidative transplants after CAR-T therapy [28]. Nevertheless, in this study, these favorable survival outcomes were achieved without transplant consolidation, which is a significant consideration for older patients who may face greater risks from such procedures. Collectively, our research highlights the effectiveness of this sequential approach in adult patients, who often have more complex disease profiles and may not be candidates for allo-HSCT.

The safety profile of sequential CAR-T cell therapy was favorable, with no treatment-related deaths reported. CRS and neurotoxicity were more common and severe after the initial CD19 CAR-T cell infusion than those following the sequential CD22 CAR-T cell infusion, which could be correlated with a higher pretreatment tumor burden before the first infusion and lower peak levels of CD22 CAR-T cells during the second infusion. Pan et al*.* also found that in pediatric populations, severe CRS and neurotoxicity were less frequent with the second infusion than those with the first infusion, indicating that the second CAR-T cell infusion tends to be better tolerated and possibly due to the patient’s immune system adapting to the initial treatment. Hematologic toxicities were the most common adverse events, but were largely manageable and reversible. The re-administration of the lymphodepletion regimen contributed to the occurrence of cytopenia. Despite a similar incidence of cytopenia between the two infusions, the need for blood product support significantly decreased during the second infusion.

EMD is evident in 15–20% of ALL relapse patients and typically connotes a marker of poor prognosis [29, 30]. Notwithstanding the innovative approach of CAR-T therapy, its efficacy in EMD is limited, which is likely ascribed to the challenges CAR-T cells face in trafficking to and persisting within extramedullary sites, compounded by the immunosuppressive microenvironment presented in these areas [31, 32]. Park et al*.* observed that patients with high disease burden and EMD had inferior median OS (12.4 months) and LFS (5.3 months) in their long-term follow-up following single CD19 CAR-T cell infusions, respectively [6]. Consistent with antecedent reports, while all patients in our cohort initially achieved CR, the long-term efficacy of sequential CAR-T cell therapy in patients with EMD was not encouraging, with shorter median OS and LFS and a higher incidence of relapse compared to those without EMD [6, 33–36]. Notably, we observed no increase in the incidence of grade 2 or higher CRS or peak CAR-T cell levels between the two infusions in patients with EMD. These findings underscore the need for additional strategies to improve responses in this domain, including radiation therapy, BITE, and consolidative allo-HSCT, to synergistically enhance CAR-T cell efficacy at extramedullary sites [37–39]. Further studies are needed to explore these potential benefits and develop optimized treatment protocols for patients with EMD.

In our cohort, relapse patterns primarily involved antigen-positive relapses, indicating that while sequential CAR-T cell therapy can effectively target leukemic cells, antigen escape mechanisms remain a significant hurdle. These findings highlight the need for additional therapeutic strategies. Emerging evidence suggests that monoclonal antibodies, such as BITE and inotuzumab ozogamicin, have demonstrated potential for durable remission in relapsed patients post-CAR-T therapy, achieving around 50–80% response rates and extending survival [40–43]. BITE has also been effective in clearing MRD before transplantation, serving as maintenance therapy post-transplant, and treating post-transplantation relapse, demonstrating excellent efficacy [44]. Additionally, in a recent phase 1 clinical trial, Srinagesh et al. demonstrated the durable efficacy and favorable safety of the combination of recombinant polymer-conjugated IL-15 receptor agonist with bispecific CD19-22 CAR-T cells. Remarkably, 89% of patients achieved MRD^−^ CR, with a 12-month LFS rate (67%) that was nearly double that of historical controls (38%) [45]. These therapies could serve as potential treatment options for patients experiencing relapse, providing a means to optimize the outcomes for these individuals.

This study has several limitations that must be acknowledged. First, the relatively small sample size from a single center and the heterogeneity of previous treatments may introduce biases that could limit the generalizability of our findings. Second, variability in the interval between sequential CAR-T cell infusions, with some patients experiencing extended intervals owing to personal health conditions, could affect treatment consistency. Furthermore, the single-arm design without a control group precludes definitive conclusions regarding the superiority of sequential therapy over dual-target approaches (co-administration infusion and tandem dual-antigen CAR-T) or sequential HSCT consolidation.

In conclusion, this study highlights the feasibility and safety of sequential CD19 and CD22 CAR-T cell therapy in adult patients with R/R B-ALL, particularly in those without transplant consolidation. Despite promising results, the risk of relapse, especially in patients with EMD, underscores the need for further research to optimize treatment strategies, including the potential role of consolidative therapies and management of antigen-positive relapses.

## Supplementary Information

Supplementary Material 1.

## Data Availability

No datasets were generated or analysed during the current study.

## Abbreviations

AEs: Adverse events
allo-HSCT: Allogeneic hematopoietic stem cell transplantation
B-ALL: B-cell acute lymphoblastic leukemia
BCA: B-cell aplasia
BITE: Bispecific T-cell engager
BM: Bone marrow
CAR: Chimeric antigen receptor
CIR: Cumulative incidence of relapse
CMV: Cytomegalovirus
CNS: Central nervous system
CNSL: Central nervous system leukemia
CR: Complete remission
CRS: Cytokine release syndrome
EMD: Extramedullary disease
HR: Hazard ratio
ICANS: Immune effector cell associated neurotoxicity syndrome
LFS: Leukemia-free survival
MRD: Minimal residual disease
NCCN: National Comprehensive Cancer Network
OS: Overall survival
PB: Peripheral blood
R/R: Relapsed or refractory
