# Supplementary material for: Prominent efficacy and good safety of sequential CD19 and CD22 CAR-T therapy in relapsed/refractory adult B-cell acute lymphoblastic leukemia
Source: Exp Hematol Oncol. 2025 Jan 3;14:2. doi: 10.1186/s40164-024-00593-5 (PMC11697943; doi:10.1186/s40164-024-00593-5)
Supplement: Supplementary file 1 — Supplementary Material 1. [file 40164_2024_593_MOESM1_ESM.pdf]

Supplementary Appendix

Contents

Supplementary Methods **3**

Clinical trial design.......... 3

Clinical procedures and assessments.......... 6

Supplementary Figure and Table **17**

Figure S1. Recovery of cytopenia in all 23 treated patients. 17

Figure S2. Peak cytokine levels in patients treated with CAR-T therapy.......... 18

Figure S3. Comparison of cytokine peak levels in patients with ≥grade 2 CRS During CD19 CAR-T vs. CD22 CAR-T Therapy. 19

Figure S4. Baseline bone marrow (BM) disease burden in patients with different grades of CRS during CD19 CAR-T therapy 20

Figure S5. Flow cytometry analysis and PET-CT scans of leukemia in relapsed patients. 21

Figure S6. Levels (A) and percentage (B) of CD19 CAR-T as well as $CD22^+$ normal B cells (C) in peripheral blood lymphocytes before CD22 CAR-T therapy. 22

Figure S7. Comparison of peak CD19 CAR-T (A) and CD22 CAR-T (B) cell levels in patients with and without extramedullary disease (EMD). 23

Table S1. Genetic risk features among 23 patients. 24

Table S2. Baseline characteristics and outcomes of patients with extramedullary disease (EMD). .. 25

Table S3. Bridging therapy regimens and post-treatment disease burden in patients at baseline. 26

Table S5. CAR-T product characteristics. 28

Table S6. Comparison of CAR-T product characteristics in patients with ongoing complete remission and relapse.......... 29

Table S7. AEs post each CAR-T cell infusion. 31

Table S8. Characteristics of relapsed patients. 33

Supplementary Methods

Clinical trial design

Overall Design

This study was a prospective, single-center design aimed at evaluating the safety and efficacy of sequential CD19 and CD22 CAR-T therapy in relapsed/refractory (R/R) B-cell acute lymphocytic leukemia (B-ALL). The selection of dose groups and enrollment numbers was based on reference data from similar trials conducted abroad. The Ethics Review Committee of the First Affiliated Hospital of Zhejiang University gave consent to this protocol.

Inclusion Criteria

Only patients who meet all the following criteria can be included in the group.

(1) Patients diagnosed with primary R/R B-ALL according to the NCCN (version 3.2020). Extramedullary diseases (EMDs) were confirmed as CD19+ and CD22+ by flow cytometry (FCM)/ immunohistochemistry (IHC) and evaluated using imaging techniques. The patients relapsed during chemotherapy, failed from re-induction chemotherapy (including the first and second generation TKIs) after relapse or had a persistent positive MRD for three months or relapsed after allogeneic hematopoietic stem-cell transplantation (allo-HSCT). Patients had positive CD19 and CD22 expression on leukaemia blasts by FCM (> 95% CD19 and CD22 positive);

(2) Age from 18 to 75 years old;

(3) Patients who relapsed after allo-HSCT, regardless of their prior treatments;

(4) Participants who voluntarily agree to participate in the trial and sign the informed consent form.

Exclusion Criteria

Participants meeting any of the following criteria will be excluded from the trial:

(1) History of epilepsy or other central nervous system disorders;

(2) Prior diagnosis of prolonged QT interval or severe cardiac diseases;

(3) Pregnant or breastfeeding;

(4) Sepsis or other uncontrolled infection;

(5) Active un controlled infections, including hepatitis B (HBsAg+), hepatitis C (PCR+) and human immunodeficiency virus;

(6) Concurrent use of systemic steroids within 2 weeks prior to treatment initiation (except for recent or current use of inhaled steroids);

(7) Previous use of any gene therapy products;

(8) Inadequate proliferative capacity in response to CD3/CD28 co-stimulation signals (<5-fold expansion);

(9) Serum creatinine >2.5 mg/dl, ALT/AST >3 times the upper limit of normal, or bilirubin >2.0 mg/dl;

(10) Presence of other uncontrolled diseases that the investigator deems unsuitable for inclusion;

(11) Any condition that the investigator believes may increase the participant's risk or interfere with trial results.

Objectives

(1) To evaluate the safety and tolerability of sequential CD19 CAR-T and CD22 CAR-T therapy in R/R B-ALL;

(2) To evaluate the efficacy of sequential CD19 CAR-T and CD22 CAR-T therapy in R/R B-ALL.

Endpoints

Primary Study Endpoints

(1) Dose-limiting toxicity (DLT);

(2) Incidence of treatment-emergent adverse events (TEAEs).

Secondary Study Endpoints

(1) overall response rate (ORR) at 1- and 3-months post-treatment per NCCN Guidelines Version 3.2020 for B-ALL;

(2) MRD-negative overall response rate within 3 months post-treatment;

(3) Duration of remission (DOR);

(4) leukemia-free survival (LFS);

(5) Overall survival (OS);

Trial Termination Criteria and Procedures

(1) Criteria for Termination from the Study

Participants who do not complete the study protocol are considered to have withdrawn early from the study. The reasons for termination (e.g., voluntary withdrawal, toxicity, death) must be documented in the case report form (CRF) and retained according to GCP requirements within the specified timeframe. Possible reasons for early termination or withdrawal include:

A. Adverse events: Participants experiencing adverse events may be withdrawn if the investigator determines that discontinuing the trial is a medical decision in the best interest of the participant.

B. Lack of efficacy: If the investigator determines that the participant is not benefiting from the study treatment and that continued participation may expose them to unforeseeable risks.

C. Major protocol violations: the participant does not meet the inclusion/exclusion criteria or fails to comply with protocol requirements.

D. Participant refusal: the participant declines further treatment or observation and voluntarily withdraws.

E. Loss to follow-up: the participant fails to return for scheduled follow-up visits and attempts to contact them are unsuccessful. Efforts to reach the participant must be

documented.

F. Death: Participant death must be recorded and reported as a serious adverse event (SAE).

G. Pregnancy: If the participant becomes pregnant, they must immediately withdraw from the study, and the event must be recorded and reported as an SAE.

H. Study termination: If the study is terminated by collaborators, the IRB, IEC, or regulatory authorities.

I. Other reasons: Any other reasons that prevent the participant from completing the study or situations where the investigator determines it is inappropriate for the participant to continue.

(2) Procedures for Withdrawal

To ensure the maximum benefit to participants, investigators may terminate the participation of individuals deemed clinically unsuitable for the study protocol or those with poor compliance. Participants may withdraw informed consent at any time during the study without any discrimination or penalty.

Clinical procedures and assessments

CD19 and CD22 CAR Construct

Humanized CD19 or CD22 CAR lentivirus was manufactured in Shanghai Yake Company under good manufacturing practices standard. T cells were collected via leukapheresis from the recipients or donors (only for patients who had undergone previous allo-HSCT and had available donor during this CAR T-cell treatment), with the goal of collecting no less than 1 x 10^9 mononuclear cells. For the majority of patients, CD19 CAR-T and CD22 CAR-T cells were prepared from the same T cell collection obtained during a single apheresis procedure. After collection, the CD19 CAR-T and CD22 CAR-T cells were manufactured sequentially. However, in a small subset of patients (n=4), the T cells collected for CD19 CAR-T preparation were insufficient to manufacture both products. In these cases, additional T cells were collected after the

infusion of CD19 CAR-T cells to prepare the CD22 CAR-T cells.

T cells were then stimulated with anti-CD3 and anti-CD28 monoclonal antibodies (GIBCO Co., Ltd.) overnight. The next day, transduction was performed at a multiplicity of infection of 5. Transduced cells were cultured in X-VIVO 15 (Lonza Co., Ltd) with 300 IU/ml interleukin-2, for the duration of cell culture.

Transduction efficiency (as the percentage of CAR^+ cell among $CD3^+$ cells) and cell viability were determined just before infusion by flow cytometry and Trypan blue exclusion respectively. Microbiological detections (Endotoxin, bacteria and mycoplasma) were conducted for each CD19 CAR-T cell and CD22 product. Five days before CAR T-cell infusion, we adopted microscopic examination, quick test and sterility test to check products from Ficoll density gradient centrifugation. Four days before CAR-T cell infusion, we utilized the same methods as Day -5 to analyze culture fluid after transduction. Two days before CAR-T cell infusion, we tested samples, fluid of which had been changed, via the same methods as Day -5. Upon infusion, samples had been cultured in a blocked environment for 48 hours, and we tested them through microscopic examination, quick test, sterility test, endotoxin test, mycoplasma test and replication-competent lentivirus test. The results of microscopic examination, endotoxin test and mycoplasma test were reported within 24 hours. The results of quick test, sterility test and replication-competent lentivirus test were reported after 5 days, 14 days and 28 days respectively. If the result was positive, the laboratory should stop cell culture and inform clinician at the same time to prepare corresponding treatment. The procedure will continue in case of negative results.

CD19 and CD22 CAR-T Cell Product Release Criteria

1. Bacterial Contamination: No bacterial growth detected.
2. Fungal Contamination: No fungal growth detected.
3. Mycoplasma Contamination: Negative.

4. Endotoxin Levels: Less than 0.25EU/ml.
5. Viable Nucleated Cells: Total count meets the investigators' requirements.
6. Proportion of Viable Cells: ≥70%.
7. $CD3^+$ T-Cell Subset in Lymphocytes: ≥80%.
8. CAR T-Cell Transduction Efficiency (Among $CD3^+$ Cells): ≥5%.
9. Residual Magnetic Beads: Fewer than $100/3\times10^6$ total cells.

Note: A small proportion of CAR T-cell products that do not fully meet the release criteria may still be infused under the treating physician's discretion if deemed necessary to prevent disease progression, provided the product is considered acceptable.

CD19 or CD22 CAR-T Cell Infusion

CD19 or CD22 CAR-T cells were sequentially administered in an inpatient setting. The required dose of CAR-T cells was calculated based on the patient's weight (kg) and the assigned dose (cells/kg), which ranged from 0.5×10^6 to 5×10^6 cells/kg. If the number of CAR-T cells manufactured fell below the minimum target dose, the infusion could proceed at the physician's discretion. CD22 CAR-T cells were administered only after achieving minimal residual disease (MRD)-negative CR or CRi following CD19 CAR-T therapy, and all adverse events related to hematologic toxicities had resolved to grade 2 or lower. Patients with grade 3 or lower leukopenia were eligible to proceed without requiring red blood cell or platelet transfusions before undergoing the second lymphodepletion. For patients with persistent grade 4 leukopenia, a prolonged infusion interval was necessary to allow recovery from cytopenia. Patients who experienced disease progression before undergoing lymphodepletion for CD22 CAR-T therapy were considered to have treatment failure and did not receive the subsequent CD22 CAR-T infusion in this trial. All CAR-T infusions were performed under the supervision of trained personnel. Patients were closely monitored during the infusion and for at least 24 hours afterward.

Evaluation Criteria for ALL Treatment Response

- CR
  1) No blast cells in peripheral blood or extramedullary leukemia;
  2) Bone marrow trilineage hematopoiesis, with blast cells <5% or <1% by flow or molecular testing;
  3) Peripheral blood ANC > 1.0×10^9/L;
  4) Peripheral blood PLT > 100×10^9/L;
  5) No recurrence within 4 weeks.
- CR with incomplete blood count recovery (CRi)

  PLT < 100×10^9/L and (or) ANC < 1.0×10^9/L. Other criteria that should meet CR. The overall response rate (ORR) = CR + CRi.
- Refractory disease

  Failure to achieve CR after induction therapy.
- Progressive disease (PD):

  The absolute number of peripheral blood or bone marrow blast cells increases by 25%, or extramedullary diseases occur.
- Disease recurrence

  Blast cells (> 5%) reappear in peripheral blood or bone marrow, or EMDs occur after a CR.

Safety Evaluation and Managements of Adverse Events (AEs)

Cytokine release syndrome (CRS)

Cytokine-related toxicity, also known as cytokine release syndrome, is caused by intense immune activation. CRS symptoms usually appear days after the T cell infusion and may appear weeks later, which can cause a wide range of symptoms and varying degrees of severity. Associated clinical symptoms include:

1) Composite symptoms: fever (may be accompanied by chills), malaise, fatigue, anorexia, muscle pain, arthralgia, nausea, vomiting, headache;

2) Skin: rash;

3) Gastrointestinal: nausea, vomiting, diarrhea;

4) Respiratory system: shortness of breath, hypoxemia;

5) Cardiovascular: tachycardia, wide pulse pressure, hypotension, increased cardiac output (early), the tendency of decreased cardiac output (late);

6) Coagulation: elevated D-dimer, hypofibrinogenemia (may be accompanied by bleeding);

7) Kidneys: azotemia;

8) Liver: transaminitis, hyperbilirubinemia;

9) Nervous system: headache, altered mental status, confusion, confusion, difficulty calling words or aphasia, hallucinations, tremors, disorders on distance discrimination, gait changes, epilepsy

10) Complications of CRS that may have fatal consequences include: cardiac dysfunction (cardiac insufficiency, respiratory cardiac arrest, malignant arrhythmia), respiratory distress syndrome in adults, neurotoxicity, renal and/or hepatic failure, cerebrovascular accident, disseminated intravascular coagulation, and other unpredictable complications.

Cytokine release syndrome (CRS) related to CAR T cells were graded according to ASTCT Consensus.

ASTCT CRS Consensus Grading

CRS Parameter	Grade 1	Grade 2	Grade 3	Grade 4
**Fever***	Temperature ≥38°C	Temperature ≥38°C	Temperature ≥38°C	Temperature ≥38°C
		With		
**Hypotension**	None	Not requiring vasopressors	Requiring a vasopressor with or without vasopressin	Requiring multiple vasopressors (excluding vasopressin)
		And/or[†]		
**Hypoxia**	None	Requiring low-flow nasal cannula[#] or blow-by	Requiring high-flow nasal cannula[#], facemask, nonrebreather mask, or Venturi	Requiring positive pressure (e.g., CPAP, BiPAP, intubation, and mechanical

			mask	ventilation)

Organ toxicities associated with CRS may be graded according to CTCAE v5.0, but they do not influence CRS grading.
** Fever is defined as temperature ≥38°C not attributable to any other cause. In patients who have CRS and receive antipyretic or anti-cytokine therapy such as tocilizumab or steroids, fever is no longer required to grade subsequent CRS severity. In this case, CRS grading is driven by hypotension and/or hypoxia.*
† CRS grade is determined by the more severe event: hypotension or hypoxia not attributable to any other cause. For example, a patient with a temperature of 39.5°C, hypotension requiring 1 vasopressor, and hypoxia requiring low-flow nasal cannula is classified as grade 3 CRS.
# Low-flow nasal cannula is defined as oxygen delivered at <6 L/min. Low flow also includes blow-by oxygen delivery, sometimes used in pediatrics. High-flow nasal cannula is defined as oxygen delivered at >6 L/min.

CRS management

1) For Grade 1 CRS:

a. Prophylactic supportive care may be used.

b. Evaluation of infection: treatment of possible fever and neutropenia, monitoring of fluid balance, and use of antipyretics and analgesics if necessary.

2) For Grade 2 CRS that occurs in non-elderly subjects without multiple complications: Prophylactic supportive care with close monitoring of cardiac and other organ function.

3) Grade 2, 3 and 4 CRS with multiple complications or elderly subjects:

a. Prophylactic supportive care may be used;

b. Anti-interleukin-6 antibody Tocilizumab (doses of 4 mg/kg and 8 mg/kg for adults and children, respectively) should be administered for more than 1 hour, and repeated administration can be considered if clinical symptoms do not resolve within 24-48 hours after administration.

c. With corticosteroid therapy as appropriate. Methylprednisolone 2mg/kg/day can be used and can be discontinued after a few days of medication. Dexamethasone (0.5 mg/kg up to 10 mg at a time) may be considered for participants with severe neurotoxic symptoms. Measures to prevent risks include eliminating contraindications, strictly following the treatment process and norms, closely monitoring your vital signs and indicators, and treating your symptoms according to the doctor's discretion.

ICANS

Neurological manifestation following CAR-T infusion are currently termed Immune Effector Cell Associated Neurotoxicity Syndrome (ICANS). In general, onset of neurotoxicity occurs within the first 2 weeks post CAR-T cell infusion. Neurologic toxicities after CAR-T therapy can include obtundation, seizures, dysphasia, and mental status changes confusion, disorientation, agitation, tremor, dysgraphia, increased intracranial pressure, papilloedema and cerebral oedema. This syndrome may arise before, during or after systemic CRS, possibly as a result of cytokines crossing the blood-brain barrier and/ or from direct cytokine production in the brain. It is possible that fever, IL-1 and IL-6 released during CRS enhances trafficking of CAR-T cells to the CSF, or it is possible that CAR-T cells traffic to recognize leukaemia or other target cells. The degree of neurotoxicity is categorized by IEC-associated neurotoxicity syndrome (ICANS) of the ASTCT Consensus while adult and child patients were separately assessed by the scale.

ASTCT ICANS Consensus Grading for Adults

Neurotoxicity Domain	Grade 1	Grade 2	Grade 3	Grade 4
**ICE score***	7-9	3-6	0-2	0 (patient is unarousable and unable to perform ICE)
**Depressed level of consciousness†**	Awakens spontaneously	Awakens to voice	Awakens only to tactile stimulus	Patient is unarousable or requires vigorous or repetitive tactile stimuli to arouse. Stupor or coma
**Seizure**	N/A	N/A	Any clinical seizure (focal or generalized) that resolves rapidly or nonconvulsive seizures on EEG that resolve with intervention	Life-threatening prolonged seizure (>5 min); or repetitive clinical or electrical seizures without return to baseline in between
**Motor findings‡**	N/A	N/A	N/A	Deep focal motor weakness such as hemiparesis or paraparesis
**Elevated ICP/cerebral**	N/A	N/A	Focal/local edema on neuroimaging§	Diffuse cerebral edema on neuroimaging;

**edema**				decerebrate or decorticate posturing; cranial nerve VI palsy; papilledema; or Cushing's triad

ICANS grade is determined by the most severe event (ICE score, level of consciousness, seizure, motor findings, raised ICP/cerebral edema) not attributable to any other cause. For example, a patient with an ICE score of 3 who has a generalized seizure is classified as grade 3 ICANS.
N/A indicates not applicable.
** A patient with an ICE score of 0 may be classified as grade 3 ICANS if awake with global aphasia, but a patient with an ICE score of 0 may be classified as grade 4 ICANS if unarousable.*
† Depressed level of consciousness should be attributable to no other cause (e.g., no sedating medication).
‡ Tremors and myoclonus associated with immune effector cell therapies may be graded according to CTCAE v5.0, but they do not influence ICANS grading.
§ Intracranial hemorrhage with or without associated edema is not considered a neurotoxicity feature and is excluded from ICANS grading. It may be graded according to CTCAE v5.0.

ICANS management

1. Formal assessment by specialist neurologist;
2. Encephalopathy assessment: Cornell Assessment of Pediatric Delirium score (CAPD, children <12 years) or Immune Effector Cell Encephalopathy score (ICE, children≥12years)
3. Imaging by CT/MRI
4. EEG
5. CSF examination
6. Blood and CSF cytokine measurements
7. IV steroid therapy or Tocilizumab may be considered for sever ICANS.

Graft Versus Host Disease (GVHD)

GVHD was graded according to European Society for Blood and Marrow Transplantation (EBMT) consensus, and needed to be discriminated with CRS. Skin, liver and intestinal rejections were evaluated according to guidelines listed in the table below. Interventions were given according to symptoms and laboratory indicators. Limited skin GVHD will be managed with topical therapies (steroid or tacrolimus

ointment). GVHD >grade II of skin, or involving liver may require prednisolone therapy (1-2mg/kg/d). GVHD could also arise if patients have previously undergone allogeneic transplant and donor derived T cells survive lymphodepletion. Donor derived GVHD arising after the allo-HSCT will be managed in line with institutional policies for transplant, but will be reported if >grade II and requiring systemic steroid therapy.

Stage	Skin	Liver	Intestinal tract
1	Maculopapular rash < 25% of body surface	Bilirubin 34-50 μmol/l	> 500 ml diarrhea/d
2	Maculopapular rash 25-50% of body surface	Bilirubin 51-102 μmol/l	> 1000 ml diarrhea/d
3	Generalized erythroderma	Bilirubin 103-225 μmol/l	> 1500 ml diarrhea/d
4	Generalized erythroderma with bullous formation and desquamation	Bilirubin > 255 μmol/l	Severe abdominal pain, with or without ileus

Grade	Degree of organ involvement
I	Stage 1-2 skin rash; no gut involvement; no liver involvement; no decrease in clinical performance
II	Stage 1-3 skin rash; stage 1 gut involvement or stage 1 liver involvement (or both); mild decrease in clinical performance
III	Stage 2-3 skin rash; stage 2-3 gut involvement or 2-4 liver involvement (or both); marked decrease in clinical performance IV
IV	Similar to Grade III with stage 2-4 organ involvement and extreme decrease in clinical performance

Other clinically common adverse reactions

Other AEs will be assessed according to the Common Terminology Standard for Adverse Events (CTCAE) version 5.0. If no CTCAE classification is present for an AE, mild, moderate, severe, and life-threatening severity will be used, corresponding to grades 1 to 4. CTCAE Grade 5 (causing death) will not be used in this study, but collected as a severity criterion; Of course, information about the death will be collected

through the death form. This entity mainly includes neutropenia, fever, hypotension, anemia, decreased plate count, leukopenia, increased AST, hypokalemia, hypophosphatemia.

Management: Most of the above conditions can be alleviated by themselves or improved after symptomatic treatment, and in rare cases may be life-threatening; Measures to prevent risks include eliminating contraindications, strictly following the treatment process, closely monitoring vital signs and indicators, and treating symptoms individually.

Pharmacokinetic studies of CAR-T cells

To fully investigate the expansion and persistence of CAR-T cells in subjects, pharmacokinetic studies will be conducted on an ongoing basis, with peak concentration, time to peak, and T-cell persistence monitored as follows:

1) Flow cytometry analysis

   Detection principle: a combination of antigen-antibody system and flow cytometric techniques. Proprietary specific CD19 or CD22 CAR T-cell detection reagent (CD19-CAR-Green and CD22-CAR-Green from Shanghai YaKe Biotechnology, Shanghai, China) could distinguish either CD19 or CD22 CAR T-cell expansion in vivo. CAR T cells/lymphocytes ratio and the absolute number. of CAR T cells (/L) in PB were calculated and samples of BM and CSF were also analyzed at the discretion of the investigator and physicians.

2) Quantitative real-time polymerase chain reaction

   CAR gene copies per ng DNA were measured using real-time quantitative PCR, with a detective threshold of 10 copies per reaction.

The monitoring of CAR-T starts 1 day before cell infusion and ends at the end of the treatment observation period, during which pharmacokinetic indexes are observed at each visit timepoint. After the survival follow-up period, the subject should also be

monitored continuously, with the endpoint of pharmacokinetic monitoring being the inability to detect CAR-T in vivo, i.e., two consecutive negative monitoring results.

Timepoints during follow-up

In principle, timepoints during follow-up are according to the following, but if the patients suffer from unexpected complications, investigators can increase the follow-up timepoints.

Timepoints and Key Assessments:

- **D-1, D0**: Vital signs, physical examination (significant changes only), cytokines, lymphocyte subsets, T-cell subsets, CAR copies, adverse events, combined medications.
- **D4, D7, D9, D11**: Similar to D-1/D0, with additional tests including blood, coagulation, biochemistry, immunoglobulins, ferritin, CRP, procalcitonin, cardiac enzymes, and viral DNA (CMV, EBV).
- **D14±2, D21±2, D28±2, D56±3**: Adds bone marrow and cerebrospinal fluid examinations, peripheral blood smears, ECG (D28), and other routine assessments. D28 specifically assesses CAR-T safety (DLT within 28 days).
- **D90±3**: Secondary endpoint to evaluate CAR-T efficacy, including remission rates and survival. Adds histopathology, ECOG scores, echocardiography, and pregnancy tests for women of childbearing age.

Early Withdrawal: Early withdrawal requires a pre-withdrawal visit within one week to complete necessary evaluations.

Survival Follow-up (Post-D90):

- Conducted every three months for two years or until CAR-T tests are negative, whichever is longer. Long-term safety follow-up extends to 15 years.
- Key data: survival time, date of death, new cancer occurrences, and adverse event monitoring.

Supplementary Figure and Table

Figure S1. Recovery of cytopenia in all 23 treated patients.

(A) Cytopenia were monitored in all 23 (100%) treated patients at different time periods. During T1, T2, T3, T5, T6, and T7, the hematological toxicity for each patient was recorded as the highest grade observed within that period. (B) Absolute lymphocyte count before the second lymphodepletion. LD, lymphodepletion. Statistical analysis was performed using the Manny Whitney test. NS, not significant.

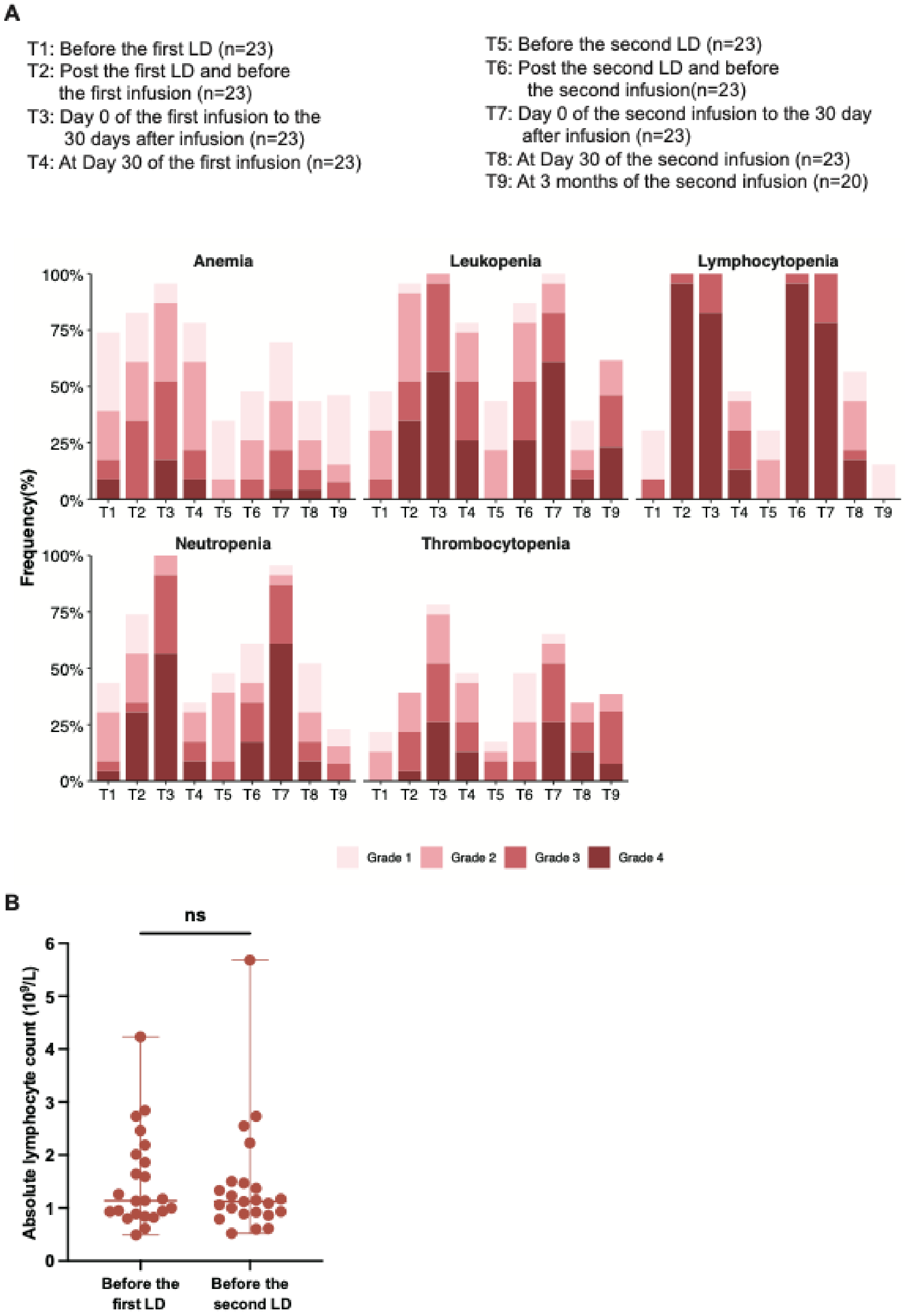

Figure S2. Peak cytokine levels in patients treated with CAR-T therapy.

(A) The peak levels of cytokines and inflammatory markers (IL-6, IL-10, IFN-γ, CRP, Ferritin, LDH) between patients with grade 0-1 cytokine release syndrome (CRS) and those with grade 2-3 CRS during CD19 CAR-T. (B) The peak levels of cytokines and inflammatory markers (IL-6, IL-10, IFN-γ, CRP, Ferritin, LDH) between patients with grade 0-1 cytokine release syndrome (CRS) and those with grade 2-3 CRS during CD22 CAR-T. Student's *t*-test or Manny Whitney test was used to compare. NS, not significant; *, *P*-value< 0.05; **, *P*-value< 0.01; ***, *P*-value< 0.001; ****, *P*-value< 0.0001.

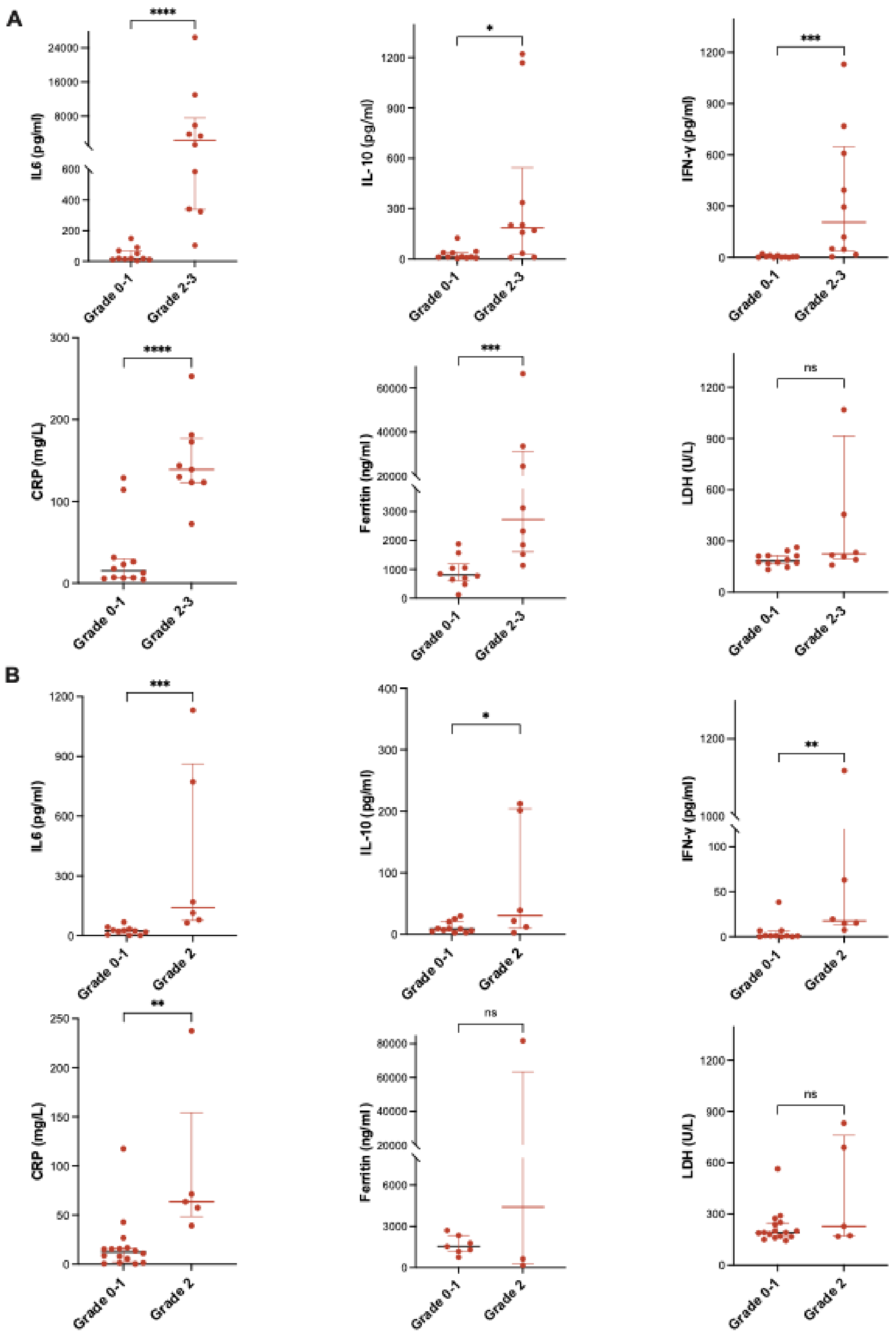

Figure S3. Comparison of cytokine peak levels in patients with ≥grade 2 CRS During CD19 CAR-T vs. CD22 CAR-T Therapy.

Student's *t*-test or Manny Whitney test was used to compare. NS, not significant; *, *P*-value< 0.05.

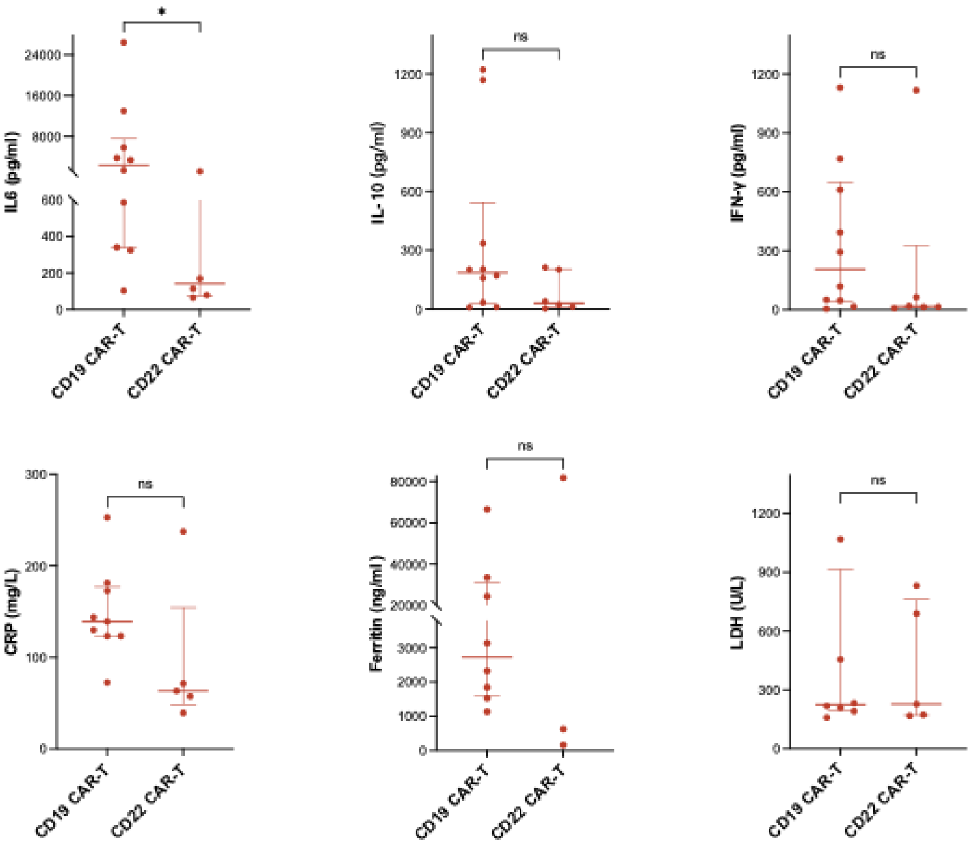

Figure S4. Baseline bone marrow (BM) disease burden in patients with different grades of CRS during CD19 CAR-T therapy

The baseline BM disease burden, represented as the percentage of blasts in BM, was assessed in patients who experienced grade ≥2 CRS compared to those with grade 0–1 CRS during CD19 CAR-T infusion. Statistical analysis was performed using the t-test, and no significant difference was observed between the two groups (P = 0.12). ns indicates no statistical significance.

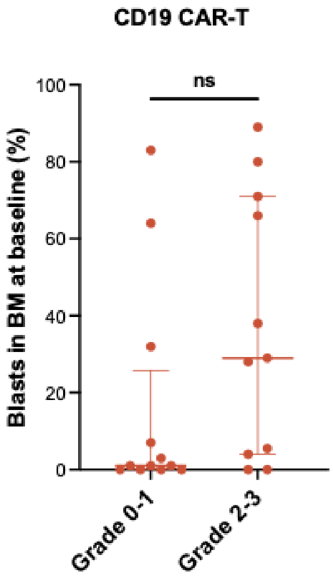

Figure S5. Flow cytometry analysis and PET-CT scans of leukemia in relapsed patients. Flow cytometry analysis of bone marrow (BM) samples from 7 relapse patients (Pt 5, 6, 7, 9, 12, 14, and 17) during relapse scenarios. Flow cytometry analysis of cerebrospinal fluid was conducted for Pt 12 with concurrent central nervous system leukemia (CNS) relapse. (B) PET-CT scans of Pt 8 showing disease status during remission after sequential CAR-T therapy and at relapse. The scans highlight the changes in metabolic activity in extramedullary disease sites before and after relapse, demonstrating the efficacy of CAR-T therapy in achieving remission and the subsequent disease recurrence in the kidney, heart, and tonsils.

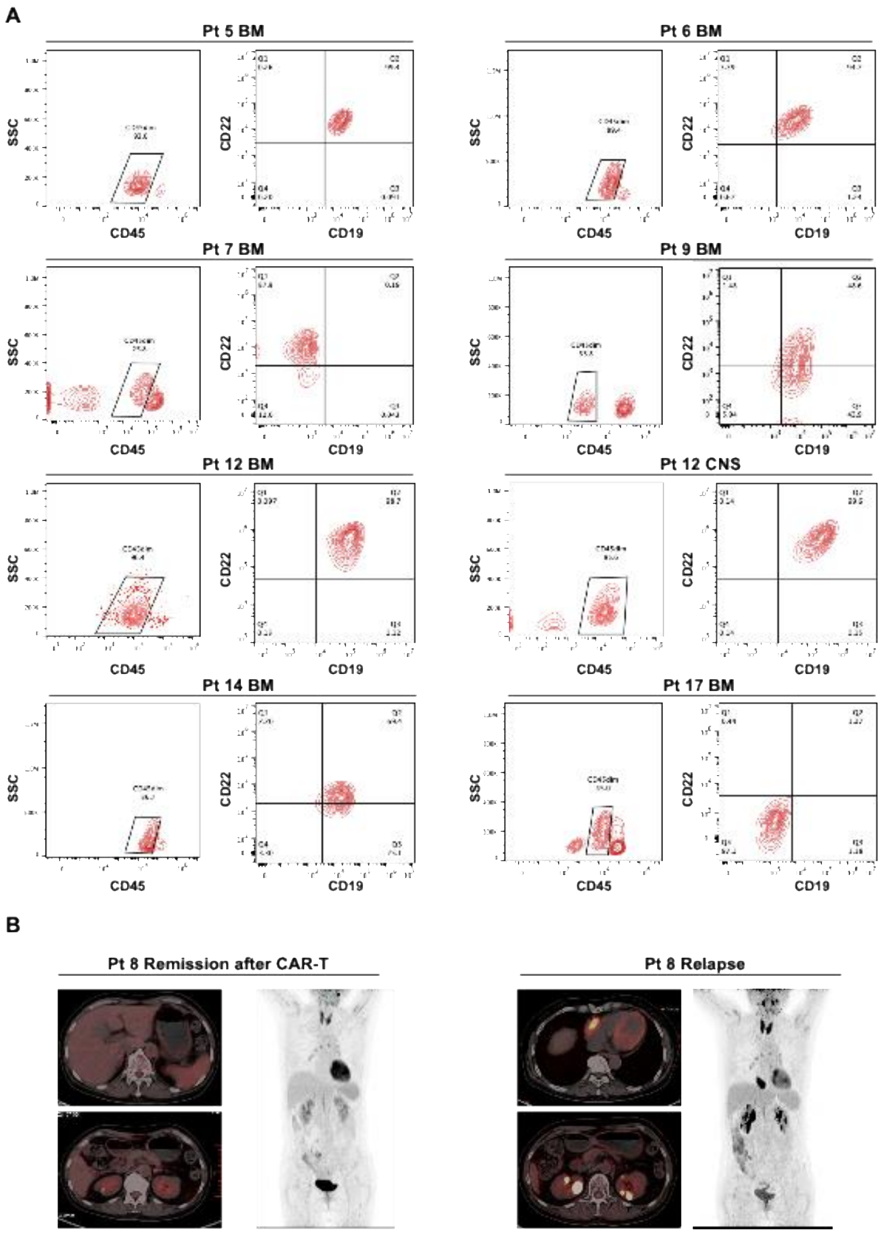

Figure S6. Levels (A) and percentage (B) of CD19 CAR-T as well as CD22+ normal B cells (C) in peripheral blood lymphocytes before CD22 CAR-T therapy.

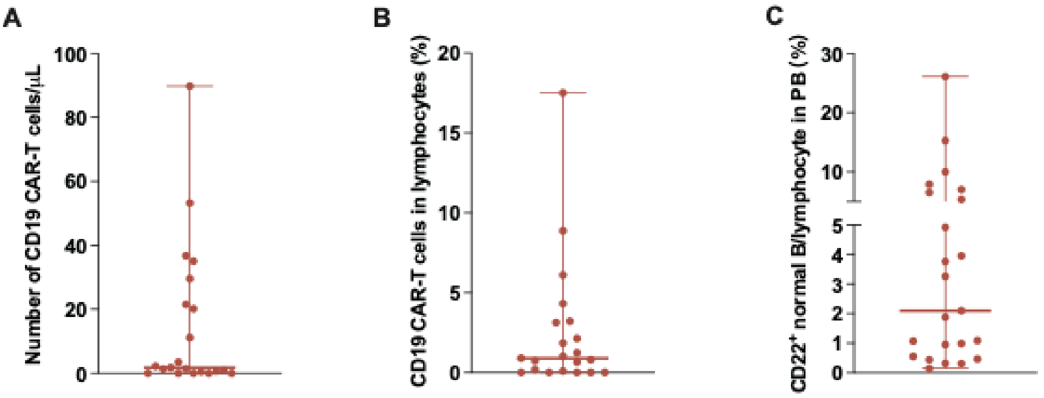

Figure S7. Comparison of peak CD19 CAR-T (A) and CD22 CAR-T (B) cell levels in patients with and without extramedullary disease (EMD).

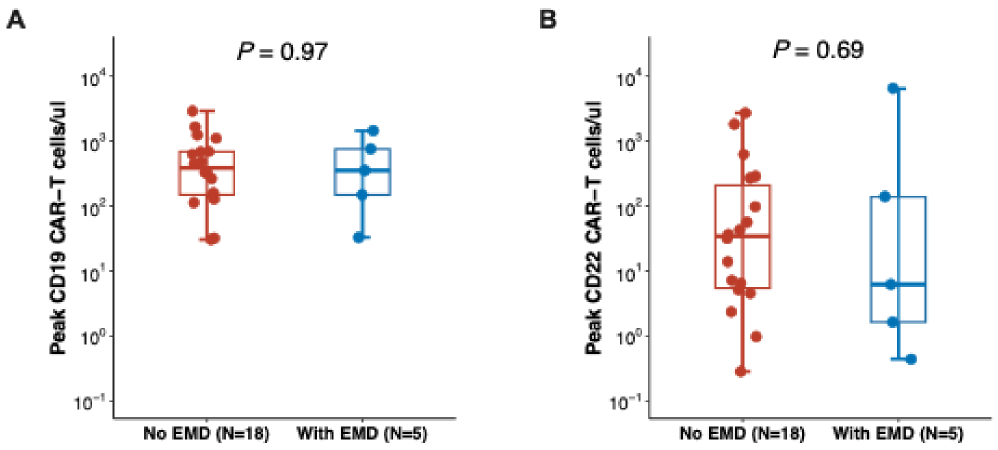

Table S1. Genetic risk features among 23 patients.

	23 Patients (%)
BCR-ABL1	4 (17.4)
IKZF1	2 (8.7)
ETV6	2 (8.7)
IDH1	2 (8.7)
PXA5	1 (4.3)
DNMT3A	1 (4.3)
TP53	1 (4.3)
SH2B3	1 (4.3)
NRAS	1 (4.3)
JAK1	1 (4.3)
FBXW7	1 (4.3)
NF1	1 (4.3)
CBLF2	1 (4.3)

Table S2. Baseline characteristics and outcomes of patients with extramedullary disease (EMD).

Patient ID	BM (%) by morphology	Sites of EMD	Disease relapse after sequential therapy (sites)	OS/LFS (months)
6	71	Uterus, fallopian tube	Yes (BM)	19.6/9.5, died of septic shock
8	0	Maxillary sinus, vagina, right rib (L4 and S1)	Yes (kidney, heart, tonsils)	38.8/9.2*
9	0	Lymph nodes, pancreas, left adrenal gland, left kidney, left ureter and multiple subcutaneous infiltration	Yes (BM)	15.2/14.4, died of disease progression
12	80	CNS	Yes (BM and CNS)	6.3/5.1, died of disease
16	1	Scalp soft tissue, lumbar neural foramen (vertebral bodies), soft tissue in the right 4 paracostal pleural cavity, left calf muscle space, stomach, liver, pancreas, penis, mediastinal lymph nodes,bone	No	4.7/4.7, died of septic shock

* Ongoing follow-up.

BM, bone marrow; CNS, central nervous system; OS, overall survival; LFS, leukemia-free survival.

Table S3. Bridging therapy regimens and post-treatment disease burden in patients at baseline.

Patient ID	Bridging therapy	Circulating blasts (%) by morphology after therapy	MRD (%) after therapy
4	Ponatinib (30mg D1-28) + Vincristine (4mg D1-8) + Dexamethasone (D1-14)	4	Negative
5	Vincristine (4mg D1) + Cyclophosphamide (0.8g D1) + Dexamethasone (D1-5)	0	0.257
17	Blinatumomab (9ug D1-6, 28ug D7-14)	4	Negative
18	Blinatumomab (9ug D1-6, 28ug D7-14)	1	Negative
19	Blinatumomab (9ug D1-6, 28ug D7-14)	0	Negative
20	Blinatumomab (9ug D1-6, 28ug D7-14)	0	Negative
21	Blinatumomab (9ug D1-6, 28ug D7-14)	1	Negative
22	Blinatumomab (9ug D1-6, 28ug D7-14)	0	Negative

MRD, minimal residual disease.

Table S4. Hematopoietic stem cell transplantation before enrollment in 10 patients.

Patient ID	Time from HSCT to CD19 CAR-T infusion (month)	Donor type	Stem-cell source	Origin of sequentially infused CAR-T cells
9	14.4	MSD	PB	Autologous*
13	72.8	MSD	PB	Donor
16	29.8	Haplo	PB	Donor
18	12.2	Haplo	PB	Donor
19	50.4	Haplo	PB	Donor
20	25.5	Haplo	PB	Donor
23	10.5	MUD	PB	Autologous*

Haplo, haploidentical donor; MSD, matched sibling donor; MUD, matched unrelated donor; PB, peripheral blood.

*The choice of autologous CAR-T cells for the two cases was due to the unavailability of donor cells at the time of CAR-T manufacturing.

Table S5. CAR-T product characteristics.

	CD19 CAR-T	CD22 CAR-T	*P* value
**CAR T-cell manufacture, median (days)**	8 (6-12)	9 (7-12)	0.12
**CAR-T cell dose, median (10^6 cells/kg)**	2.1 (0.8-3.6)	2.1 (1.1-3.0)	0.93
**CAR-T cell viability (%)**	91.9 (80.7-95.5)	91.1 (81.1-96.2)	0.88
**CAR transduction rate (%)**	59.7 (10.6-77.9)	48.1 (20.7-70.0)	0.01
**CD4 CAR-T/CAR-T ratio**	85.6 (71.0-97.4)	69.8 (34.6-91.8)	<0.001
**CD8 CAR-T/CAR-T ratio**	11.5 (1.9-25.1)	26.1 (5.0-61.8)	<0.001
**CD4 /CD8 CAR-T ratio**	7.4 (2.8-52.1)	2.7 (0.6-18.3)	0.02

Table S6. Comparison of CAR-T product characteristics in patients with ongoing complete remission and relapse.

	Ongoing CR (N=15)	Relapse (N=8)	*P* value
**CD19 CAR-T, median(range)**			
Manufacture day	8 (6-10)	8.5 (6-12)	0.29
Dose, 10^6 cells/kg	2.1 (1.0-2.9)	2.1(0.8-3.6)	1.00
Cell viability, %	91.3 (80.7-95.5)	92.9 (88.6-94.9)	0.07
CAR transduction rate, %	59.7 (43.3-77.4)	58.5 (10.6-77.9)	0.73
CD4 ratio, %	85.7 (71.0-97.4)	85.5 (77.0-93.8)	0.55
CD8 ratio, %	12 (1.9-25.1)	11.4 (4.3-17.5)	0.45
CD4/CD8 ratio	7.1 (2.8-52.1)	7.5 (4.4-21.7)	0.68
**CD22 CAR-T, median (range)**			
Manufacture day	9 (7-12)	9.5 (7-11)	0.57
Dose, 10^6 cells/kg	2.2 (1.7-3.0)	1.8 (1.1-2.5)	0.09
Cell viability, %	91.6 (85.4-96.2)	90.7 (81.1-94.5)	0.58
CAR transduction rate, %	48.1 (25.3-70.0)	45.2 (20.7-64.1)	0.42
CD4 ratio, %	64.7 (34.6-89.9)	77.4 (57.5-91.8)	0.09
CD8 ratio, %	30.2 (5.1-61.8)	19.8 (5.0-36.8)	0.11

CD4/CD8 ratio	2.2 (0.6-17.6)	4.3 (1.6-18.3)	0.17

CR, complete remission.

Table S7. AEs post each CAR-T cell infusion.

	CD19 CAR-T					CD22 CAR-T				
	All	Grade 1	Grade 2	Grade 3	Grade 4	All	Grade 1	Grade 2	Grade 3	Grade 4
**Hematological**										
Leukopenia	23 (100)	0	1 (4.3)	9 (39.1)	13 (56.5)	23 (100)	1 (4.3)	3(13.0)	5 (21.7)	14 (60.9)
Neutropenia	23 (100)	0	2(8.7)	8 (34.8)	13 (56.5)	22 (95.7)	1 (4.3)	1 (4.3)	6 (26.1)	14 (60.9)
Lymphopenia	23 (100)	0	0	4 (17.4)	19 (82.6)	23 (100)	0	0	5 (21.7)	18 (78.3)
Anemia	22 (95.7)	2(8.7)	8 (34.8)	8 (34.8)	4 (17.4)	16 (69.6)	6 (26.1)	5 (21.7)	4(17.4)	1 (4.3)
Thrombocytopenia	18 (78.3)	1 (4.3)	5 (21.7)	6 (26.1)	6 (26.1)	15 (65.2)	1 (4.3)	2(8.7)	6 (26.1)	6 (26.1)
**CRS**	18 (78.3)	7 (30.4)	9 (39.1)	2 (8.7)	0	9 (39.1)	3 (13.0)	6 (26.1)	0	0
**ICANS**	2 (8.7)	0	2 (8.7)	0	0	0	0	0	0	0
**Gastrointestinal disorders**										
Abdominal distension	2 (8.7)	1 (4.3)	1 (4.3)	0	0	1 (4.3)	1 (4.3)	0	0	0
Diarrhea	4 (17.4)	3 (13.0)	1 (4.3)	0	0	2 (8.7)	2 (8.7)	0	0	0
Vomiting	0	0	0	0	0	0	0	0	0	0
Abdominal pain	3 (13.0)	3 (13.0)	0	0	0	0	0	0	0	0
Dry mouth	2 (8.7)	2 (8.7)	0	0	0	0	0	0	0	0
Nausea	4 (17.4)	4 (17.4)	0	0	0	4 (17.4)	3 (13.0)	1 (4.3)	0	0
**Metabolism and nutrition disorders**										
Hypokalaemia	14 (60.9)	7 (30.4)	6 (26.1)	1 (4.3)	0	13 (56.5)	8 (34.8)	4 (17.4)	1 (4.3)	0
Hypocalcaemia	15 (65.2)	9 (39.1)	6 (26.1)	0	0	8 (34.8)	6 (26.1)	1 (4.3)	1 (4.3)	0

Hypoalbuminaemia	19 (82.6)	15 (65.2)	4 (17.4)	0	0	15 (65.2)	14 (60.9)	1 (4.3)	0	0
Pruritus	2 (8.7)	2 (8.7)	0	0	0	4 (17.4)	2 (8.7)	2 (8.7)	0	0
Rash	1 (4.3)	0	1 (4.3)	0	0	1 (4.3)	0	1 (4.3)	0	0
**Respiratory diseases**	0	0	0	0	0	0	0	0	0	0
Productive cough	3 (13.0)	3 (13.0)	0	0	0	3 (13.0)	3 (13.0)	0	0	0
**Other**										
Prolonged APTT	2 (8.7)	1 (4.3)	1 (4.3)	0	0	1 (4.3)	1 (4.3)	0	0	0
Increased GGT	7 (30.4)	3 (13.0)	2 (8.7)	2 (8.7)	0	6 (26.1)	2 (8.7)	1 (4.3)	3 (13.0)	0
Increased INR	2 (8.7)	2 (8.7)	0	0	0	0	0	0	0	0
Insomnia	1 (4.3)	1 (4.3)	0	0	0	2 (8.7)	2 (8.7)	0	0	0
Hypertension	0	0	0	0	0	0	0	0	0	0
Fatigue	8 (34.8)	6 (26.1)	2 (8.7)	0	0	4(17.4)	3 (13.0)	1 (4.3)	0	0
Dizziness	7 (30.4)	7 (30.4)	0	0	0	2 (8.7)	2 (8.7)	0	0	0

AE, adverse event; CAR-T, chimeric antigen receptor-T; CRS, cytokine release syndrome; ICANS, immune effector cell-associated neurotoxicity syndrome; APTT, activated partial thromboplastin time; GGT, gamma-glutamyl transferase; INR, international normalized ratio.

Table S8. Characteristics of relapsed patients.

Pt	Prior EMD involvement	Relapse type	Blasts/MRD in BM	Immunophenotype at baseline	Immunophenotype at relapse	OS/LFS (months)
5	No	BM	82/67.9	$CD19^{+}CD34^{+}CD10^{+}CD38^{dim}CD22^{+}$ $CD20^{dim}CD58^{dim}CD81^{dim}CD45^{dim}$	$CD19^{+}CD34^{+}CD10^{+}CD38^{+}CD22^{+}$ $CD20^{-}CD58^{+}CD81^{-}CD45^{dim}$	44.4/20.8*
6	Yes	BM	78/66.1	$CD19^{+}$ $CD34^{+}CD10^{+}$ $CD38^{dim}CD22^{+}$ $CD15^{+}cyCD79a^{+}nTdT^{+}$	$CD19^{+}CD34^{+}CD10^{+}CD38^{dim}$ $CD22^{+}CD20^{-}CD58^{+}CD81^{+}CD45^{dim}$	19.6/9.5, died of septic shock
7	No	BM	58/51.8	$CD19^{+}CD34^{dim}CD10^{part}CD38^{+}CD22^{dim}$ $CD20^{part}CD58^{dim}CD81^{part}CD45^{dim}$	$CD19^{+}CD34^{part}CD10^{+}CD38^{dim}CD22^{+}$ $CD20^{part}CD58^{+}CD81^{+}CD45^{dim}$	16.8/7.7, died of HSCT-related complications
8	Yes	Extramedullary	2/Neg	/	/	38.8/9.2*
9	Yes	BM	28/27.3	$CD19^{+}$ $CD34^{+}CD22^{+}CD20^{+}$ $CD24^{+}$ $CD45^{dim}$	$CD19^{+}CD34^{+}CD10^{+}CD38^{dim}CD22^{+}$ $CD20^{-}CD58^{+}CD81^{+}CD45^{dim}$	15.2/14.4, died of disease progression
12	Yes	BM and extramedullary (CNS)	89/79.9	$CD19^{+}CD34^{+}CD10^{+}CD22^{+}$ $CD200^{+}cyCD79a^{+}nTdT^{+}$	$CD19^{+}CD34^{part}CD10^{+}CD38^{dim}CD22^{dim}$ $CD20^{dim}CD58^{-}CD81^{+}CD45^{dim}$	6.3/5.1, died of disease progression

14	No	BM	71/66/6	$CD19^{+}CD34^{-}CD10^{dim}CD38^{+}CD22^{+}CD20^{-}CD58^{+}CD81^{+}CD45^{dim}$	$CD19^{+}CD34^{part}CD10^{+}CD38^{+}CD22^{+}CD20^{-}CD58^{+}CD81^{+}CD45^{dim}$	12.1/6.7, died of disease progression
17	No	BM	21/2.8	$CD19^{+}CD34^{part}CD10^{+}CD38^{dim}CD22^{+}CD20^{-}CD58^{+}CD81^{-}CD45^{dim}$	$CD19^{-}CD34^{part}CD10^{-}CD38^{+}CD22^{-}CD20^{-}CD58^{+}CD81^{-}CD45^{dim}$	10.8/5.4*

* Ongoing follow-up.

BM, bone marrow; CNS, central nervous system; HSCT, hematopoietic stem cell transplantation; LFS, leukemia-free survival; MRD, minimal residual disease; Neg, negative; OS, overall survival; /, not applicable.
